# Monitoring of cocoa post-harvest process practices on a small-farm level at five locations in Ecuador

**DOI:** 10.1016/j.heliyon.2022.e09628

**Published:** 2022-06-09

**Authors:** Stefanie Streule, Susette Freimüller Leischtfeld, Martina Galler, Susanne Miescher Schwenninger

**Affiliations:** a Zurich University of Applied Sciences (ZHAW), Institute of Food and Beverage Innovation, Food Biotechnology Research Group, Einsiedlerstrasse 31, 8820 Wädenswil, Switzerland; b Lindt Chocolate Competence Foundation, Seestrasse 204, 8802 Kilchberg, Switzerland

**Keywords:** Cacao nacional, Cocoa bean fermentation, Fermentation techniques, Fermentation in bags, Sensory description

## Abstract

Cocoa post-harvest practices were monitored on a small-farm scale (ca. 50 kg fresh beans) at five intermediaries from four provinces in Ecuador: (A) in Manabí, (B) and (E) in Los Ríos, (C) in Cotopaxi, (D) in Guayas. Temperature, pH (pulp, cotyledon), cell counts (yeasts, lactic acid bacteria, acetic acid bacteria) were recorded daily, and cut-tests and sensory descriptive analysis evaluated end quality. An overall inconsistency and variability in processing were observed with different fermentation devices (jute/plastic bags, wooden boxes), pre-drying, turning during fermentation, fermentation duration, and different drying processes (temperatures, direct/indirect). Key parameters (maximum temperature, pH cotyledon development) revealed a significant impact of the fermentation device on the post-harvest process and, therefore, on the fermentation development. 67–74 h in jute bags without turning was sufficient to reach well-fermented cocoa beans without moldy off-flavors, whereas 133 h in plastic bags without turning resulted in 3 ± 1% moldy beans and cocoa liquor with moldy off-flavor. Drying at high temperatures (80 ± 10 °C) with direct heat contact resulted in beans roasted to burnt off-flavor. Conclusively, the whole post-harvest process was crucial for well-fermented beans without off-flavor. Plastic bags seemed unsuitable, while jute bags could be an alternative to wooden boxes.

## Highlights

• Common fermentation devices in Ecuador: Jute bags, plastic bags, wooden boxes.
• High variability in overall post-harvest processing in Ecuador.
• Fermentation in jute bags could be an alternative to wooden boxes.
• Fermentation in plastic bags without turning seemed not suitable.

## Graphical abstract

**Figure:**
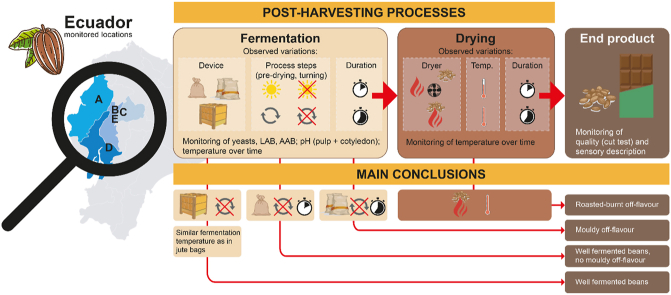

## 1 Introduction

Today, Ecuador is the third-largest cocoa producer worldwide (ICCO, 2021). It is known for its fine flavor cocoa (named “Cocoa Nacional” or “Arriba”) and counts as one of the major fine flavor cocoa exporters in terms of volumes (Anecacao, 2015a; ICCO, 2019). Cocoa Nacional has a full cocoa flavor with additional strong floral and spicy flavors (Badrie et al., 2015; Fowler, 2009) if fermented and dried well. Otherwise, the flavor potential is not reached, and the beans are traded as bulk cocoa, which is also the classification of CCN-51, the second variety in Ecuador that is characterized by disease-tolerance combined with high productivity and quality (Anecacao, 2015b; Fowler, 2009; ICCO, 2019).

The start of the chocolate supply chain, the post-harvest processing of the raw beans, strongly influences the characteristics of its end, the chocolate (Saltini et al., 2013). After harvesting, the pods are opened, followed by the immediate start of a spontaneous fermentation where yeasts, lactic (LAB), and acetic acid bacteria (AAB) degrade the sugars of the mucilaginous pulp to ethanol and acids concomitant with heat development. The diffusion of microbial metabolites into the cotyledon triggers biochemical transformation resulting in the production of aroma precursors and reduction of bitterness and astringency, and death of the embryo (Schwan and Wheals, 2004). After fermentation, the beans are dried to the humidity of 6–8% (Thompson et al., 2001). Factors such as type of cocoa, climatic conditions and growth location (Afoakwa, 2010), ripeness of the cocoa pods and pod storage (Amoa-Awua, 2015; Camu et al., 2007; Hinneh et al., 2018; Sulaiman and Yang, 2015), batch sizes during fermentation (Camu et al., 2007), fermentation time (Hamdouche et al., 2019; Portillo et al., 2011), turning during fermentation (Camu et al., 2008; Dircks, 2009), or fermentation device (Quevedo Guerrero et al., 2018) influence cocoa quality.

Worldwide, cocoa post-harvest processing is mainly done by smallholder farmers under rural conditions (Muñoz et al., 2019; Purcell, 2018; Thompson et al., 2001). Farmers grow, harvest, ferment, and dry the cocoa beans in a non-industrialized way without controlling the processing conditions (Muñoz et al., 2019; Saltini et al., 2013). In Ecuador, the individual farmers sell the beans partially fermented or partially or fully dried to local intermediaries where, if necessary, they are fermented or dried to the end before selling to exporters (Middendorp et al., 2020). The varying post-harvest practices combined with the mixing of beans originating from different process stages and various farmers and intermediaries result in very heterogeneous batches of cocoa beans favoring low quality (Muñoz et al., 2019; Saltini et al., 2013).

Worldwide, fermentation techniques applied are diverse and incorrect handling, especially during spreading the beans at day and heaping at night, resulting in under-fermented cocoa with an increased risk of mold growth and off-flavors (Afoakwa, 2014; Papalexandratou et al., 2011b; Wood and Lass, 1985). Typical practices in Ecuador are fermenting in wooden boxes, jute bags, and plastic tubs (Rivera Fernández et al., 2012).

Considering the high variations of cocoa bean processing in Ecuador, our study closely monitored representative post-harvest techniques at five intermediaries. This was achieved by field experiments (small-farm scale) conducted according to the instructions of each intermediary. This was accompanied by temperature measurements, physicochemical (pH value of pulp and cotyledon) and microbiological (enumeration and identification of yeasts, lactic acid bacteria, acetic acid bacteria) analyses. Quality assessment of fermented and dried cocoa beans (cut-test, sensory description) allowed to evaluate potential influences of the varying processes on the overall bean quality.

## 2 Material and methods

### 2.1 Post-harvest trials and sampling

Post-harvest processes (fermentation, drying) were performed and monitored at five intermediaries in Ecuador between 06/2017 and 01/2018: (A) province Manabí (n = 3 independent runs x 4 dependent repetitions each), (B) and (E) Los Ríos (n = 2 × 4 at B; n = 2 × 2 at E in plastic bags (EP) + 2 × 2 in wooden boxes (EW), respectively), (C) Cotopaxi (n = 3 × 4), (D) Guayas (n = 2 × 4 + 1 × 3).

Cocoa Nacional, selected according to phenotypical characteristics as pod color development during ripening, pod shape and apex form, pod surface rugosity, and furrows, was used in this study. Cocoa pods were harvested from approx. 25–30 and 60–80 years old trees in province Guayas and Manabí, Los Ríos and Cotopaxi, respectively, by one to twelve farmers per intermediary. After harvesting, the pods were opened on day 0 (pod storage 0–1 day). The extracted healthy beans were transported in plastic bags/buckets to the intermediary and were divided into two to four jute (capacity: approx. 115 kg wet cocoa beans, e.g., Figure 3II) or plastic bags (capacity: approx. 50 kg wet cocoa beans, e.g., Figure 3IV) or wooden boxes (1.3 m length x 0.63 m width x 0.57 m height, Figure 3V) with approx. 50 kg for each dependent repetition. Visually clean jute and plastic bags (prior used for storage of semi-dried/dried cocoa beans) were used at every fermentation run at location A, B, C, E. Jute bags at location D and the wooden box at location E have already been used before these trials, hence dried pulp residues of prior fermentations adhered on the devices. Fermentation and drying were carried out according to the respective intermediary's practices.

**Figure 1:**
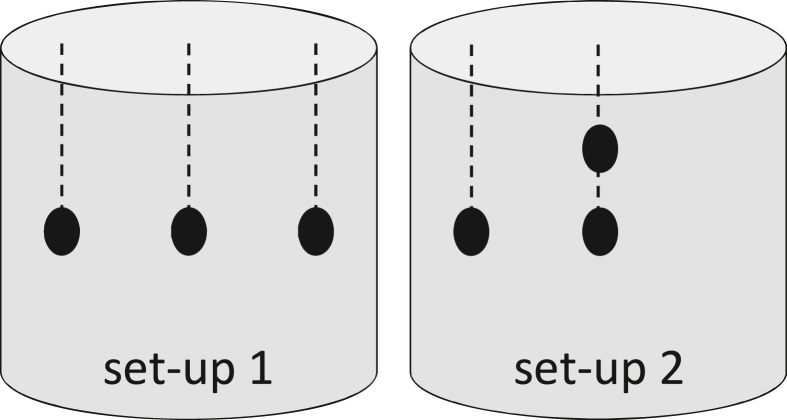
Position of the three temperature probes per dependent repetition and sample taking during fermentation; Position in set-up 1 for location A (run A1, A2), location B (run B1, B2), location C (run C1), location D (run D1) and position in set-up 2 for location A (run A3), location C (run C2, C3), location D (run D2, D3), and location E (run E1, E2).

Samples were taken daily during fermentation from different points by hand using disinfected gloves according to the temperature probe positions (set-up 1 and set-up 2, Figure 1). The mixed samples were transported under uncooled conditions for a maximum of 45 min before determining microbial counts, pulp and cotyledon pH of fresh and fermenting beans, and cut-tests of dried beans. Dried beans were stored and transported to Switzerland for sensory description.

### 2.2 Analyses

#### 2.2.1 Measurement of temperature

The temperature of bean-pulp-mass was recorded every 15 min with a datalogger analogous to Romanens et al. (2018). During fermentation, three probes per dependent repetition were positioned according to Figure 1 (set-up 1 for run A1, A2, B1, B2, C1, D1; set-up 2 for A3, C2, C3, D2, D3, E1, E2). Three probes per repetition were positioned randomly on the floor and covered with beans during sun-drying. Similarly, one probe was placed randomly in heaped beans in the dryer during artificial drying.

#### 2.2.2 Enumeration, isolation, and MALDI-TOF MS identification of microorganisms

For the enumeration of yeasts, LAB, and AAB, 10 g of cocoa beans were mixed with 90 ml diluent (0.1% bacteriological peptone, HiMedia; 0.85% sodium chloride, Sigma-Aldrich; [w/v]) and manually kneaded for 2 min. Serial dilutions were either plated or analyzed by a drop-plate method (Romanens et al., 2018) with 0.01 ml of diluted sample on yeast glucose chloramphenicol medium (YGC, Sigma-Aldrich) for yeasts and on yeast peptone mannitol medium (YPM), supplemented with cycloheximide and penicillin (Romanens et al., 2018) for AAB. Both were incubated aerobically at 26 °C for 2–4 days; LAB were count on De Man Rogosa Sharp medium (MRS containing Tween 80 and 0.004% cycloheximide (all Sigma-Aldrich) diluted in water) and incubated anaerobically at 37 °C for 2–3 days.

After colony growth, 5 colonies were randomly selected per day and medium. These isolates were purified on an agar medium, transferred to slant agar in a Cryotube, incubated for 1 day, and sent to Switzerland. As shown above, growth conditions were applied according to the respective microbial group. In Switzerland, confirmation assays were performed (Romanens et al., 2018), which resulted in a factor that was considered in the calculation formula for the CFU (colony forming units) per gram according to ISO 7218:2007.

A total of 886 isolates (315 taken from YGC, 278 from MRS, 293 from YPM) was identified by MALDI-TOF MS in Switzerland (Miescher Schwenninger et al., 2016).

#### 2.2.3 Measurement of pH of cotyledon and pulp

PH cotyledon and pulp were determined using indirect methods (Romanens et al., 2018, 2020).

#### 2.2.4 Cut-test and sensory description of dried cocoa beans

Dried cocoa beans were evaluated by experienced people by a cut-test by cutting 3 × 100 beans lengthwise to expose a maximum cotyledon surface allowing classification by the attributes well-fermented, slightly fermented, violet, slaty, moldy, or insect-infested. Total fermented beans were defined as the sum of the well and slightly fermented beans.

For the sensory evaluation, a mixed sample of the dependent repetitions per independent run of each location was used for cocoa liquor production. For location E, samples of the two fermentation devices per run were also mixed (E1 = E1P + E1W and E2 = E2P + E2W). The dried beans were stored at least for 6 weeks aiming at stabilization of acidity followed by roasting at 121 °C for 25 min (piece weight 80–128 g/100 beans; humidity 6.5–7.8%) with slight adjustments for out-of-range bean sizes (shorter time for smaller beans, the lower temperature during the longer time for bigger beans) and varying moisture contents (increasing humidity resulted in increasing roasting time). Then, beans were broken to nibs (Cocoa Breaker 240-1-50, Capco Test Equipment), pealed, first mechanically (Cocoa Winnower 240-1-50, Capco Test Equipment) and then manually to ensure the removal of the maximum amount of skins. The pealed nibs were ground for approx. 4 h (stone mill, CocoaTown Melanger) until cocoa liquor with a particle size <16 μm was reached (approx. 4 h milling). The finished liquor was stored at 18 °C for a minimum of one week, aiming at aroma stabilization before sensory evaluation. For the sensory evaluation, sample portions of 2 g were filled in covered plastic cups and melted at 35 °C shortly before tasting.

The samples were evaluated regarding flavor (basic tastes, retronasally perceived aromas, and astringency) upon tasting.

A generic descriptive analysis based on QDA (Lawless and Heymann, 2010) was carried out by the trained sensory panel (n = 10) of Lindt (Kilchberg, Switzerland). Prior to degustations, panelist were informed about the scope of the study and informed consent was obtained from all participants in the sensory experiments. Samples underwent quality control to ensuring adherence to food safety standards. The anonymized setup ensured the observation of the individuals privacy rights.

The attribute list for the samples was defined in pre-trial sessions using an individual sorted napping task coupled with word association (described, e.g., in Lê et al., 2015) followed by a reduction and definition based on subsamples that stretched the sensory space. The defined attributes were: acidity, bitterness, fruity:citrus, fruity:banana, floral, nutty, woody, spicy, malty, roasted-burnt, earthy/moldy, chemical, astringency. Panelists trained flavor recognition with references mentioned in Table 1 under “Definitions”. E.g. for the earthy/moldy off-flavor wet earth and white mold cheese were filled in opaque bottles and was smelled, while for basic tastes and astringency, water solutions were tasted. The profiling was performed once sample discrimination and repeatability as well as panel agreement were sufficiently high. The panelists rated each sample in duplicate on a 10-cm scale for the data collection (scale in Table 1).

**Table 1:** Definition of flavor attributes and scales.

Attribute	Definition	Scale
Acidity	Basic taste, triggered e.g. by citric, acetic or lactic acid solution	Very weak – very strong
Bitterness	Basic taste, triggered e.g. by caffeine solution	
Fruity:citrus	Aroma reminding of citrus fruits such as lemon or orange	
Fruity:banana	Aroma reminding of banana (dried)	
Floral	Aroma reminding of dried flowers (hay flower) or tea (orange blossom) or orange blossom soap	
Nutty	Aroma reminding of unroasted nuts with or without skins as well as raw cocoa bean (without pungent acidity)	
Woody	Aroma and mouthfeel reminding of wet wood (wooden ice cream sticks)	
Spicy	Aroma reminding of pepper or tobacco	
Malty	Aroma reminding of malt powder dissolved in water	
Roasted-burnt	Aroma reminding of toasted/slightly burnt cacao nibs or burnt bread	Not roasted – roasted - burnt
Earthy/moldy	Aroma that is musty and reminding of wet earth, and white mold cheese	Very weak – very strong
Chemical	Aroma reminding of medicine (aerosol plasters) or chlorine (Javel water)	
Astringency	Dry, rough or furry mouthfeel on the tongue and palate, triggered e.g. by alum solution or tannin solution	

#### 2.2.5 Statistics

For all analyses besides sensory description, mean values of the two to four dependent repetitions per independent fermentation run were calculated. The processes of the five intermediaries were treated separately. The standard deviation and or confidence interval was represented as an error bar when n ≥ 3. Whenever appropriate, ANOVA was applied, followed by posthoc (e.g., Tukey) and multivariate data analysis (PCA). Excel version 2008 and R version 3.5.2 were used. In the case of sensory description, a mixed ANOVA was implemented with PanelCheck version 1.4.2. Data were assumed to be normally distributed, and a significant value of p < 0.05 was used.

## 3 Results

### 3.1 Variations of fermentation and drying processes

**Figure 2:**
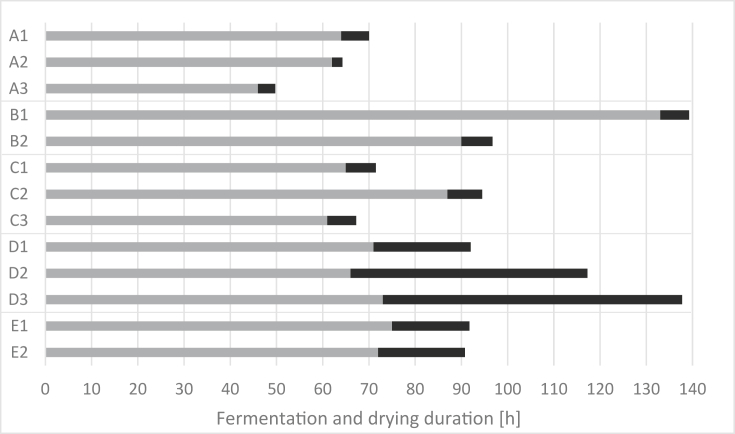
Duration [h] of fermentation (light grey) and drying in an artificial dryer (dark grey) at the five locations A (run A1, A2, A3), B (run B1, B2), C (run C1, C2, C3), D (run D1, D2, D3), E (run E1, E2).

**Figure 3:**
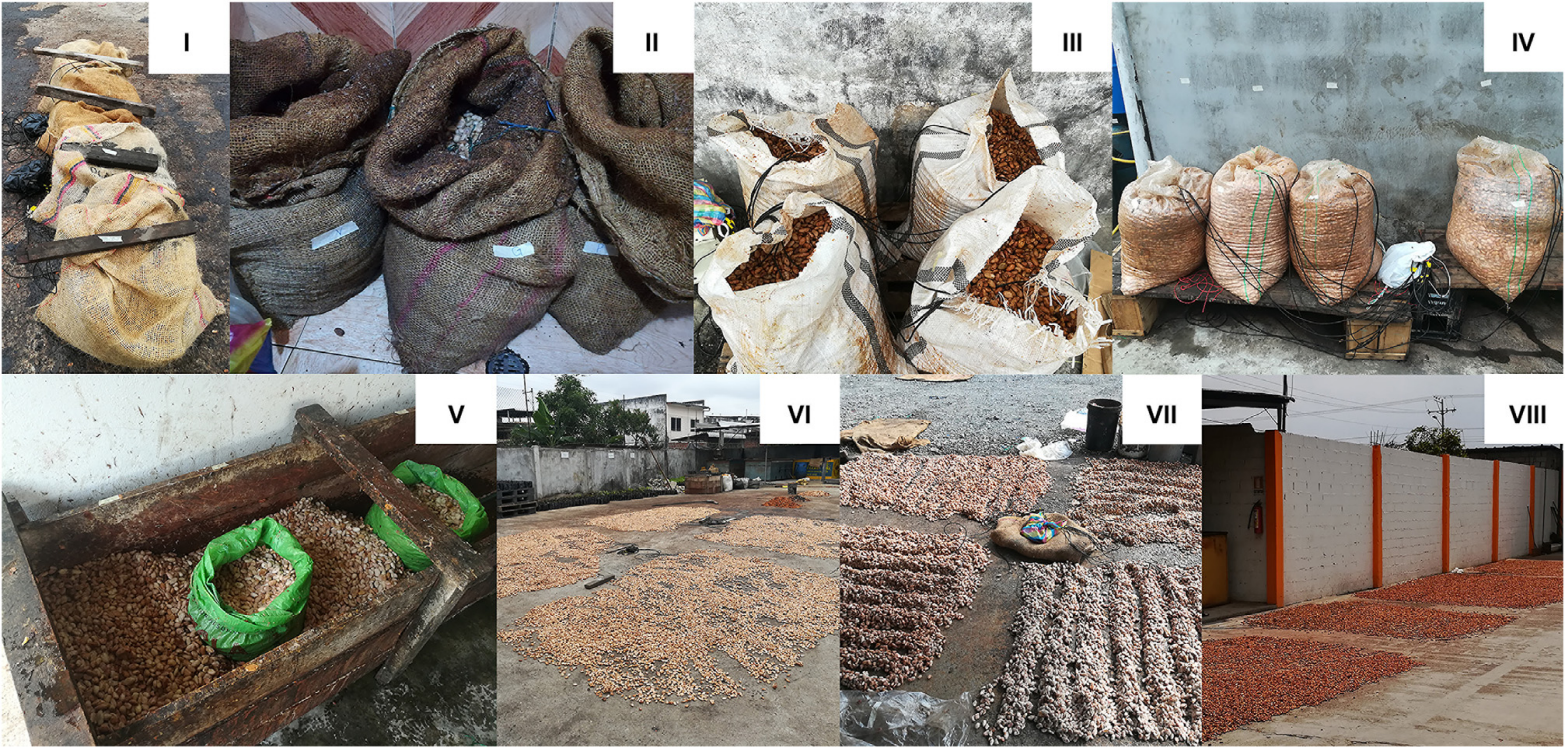
Fermentation during night in jute bags at day 2, location A (I); Fermentation in jute bags, location D (II); Fermentation in plastic bags, location B (III) and C (IV); Fermentation in wooden boxes and in plastic bags, location E (V); Pre-drying during day 1, location A (VI); Pre-drying at day 1, location D (VII); Pre-drying at day 3, location E (VIII).

Cocoa beans were fermented and dried according to the respective intermediary, with the highest differences in the fermentation device, process steps such as pre-drying in the sun and turning, drying (Table 2), and fermentation and drying time (Figure 2). The main used devices were jute bags (location A, Figure 3I; D, Figure 3II), plastic bags (location B, Figure 3III; C, Figure 3IV; E, Figure 3V), and wooden boxes (location E, Figure 3V). Pre-drying during fermentation was done at location A (Figure 3VI) and D, at the latter, beans with low pulp content at day 0 were filled directly into a device; otherwise, beans were pre-dried first (Figure 3VII). Pre-drying during fermentation was done by spreading the beans on a concrete floor. Beans were not turned at locations B (except B1 where the beans were fermented with the placenta that was removed on day 3 while heaping the beans on the concrete floor followed by removing the placenta manually and putting the beans back into the same bags, which resulted in a turning step), C, D and E. One day before artificial drying, the beans were pre-dried at B and C and stored in plastic bags (B) overnight or in the switched-off dryer (C). At locations A, D, and E (Figure 3VIII), pre-drying was done on the day of artificial drying. At Location A, beans fermented the significant fastest with 62–64 h (A1, A2) and 46 h (A3) compared to B, C, E (p < 0.05). Beans of run B1 were fermented significantly longer (p < 0.05) up to 133 h (90 h for B2).

**Table 2:** Fermentation techniques (device, pre-drying during fermentation, turning during fermentation, pre-drying prior to artificial drying, artificial drying) at the five locations A (run A1, A2, A3), B (run B1, B2), C (run C1, C2, C3), D (run D1, D2, D3), E (run E1W, E2W, E1P, E2P); main device in bold.

Location	Run	Fermentation device	Pre-drying during fermentation	Turning during fermentation	Pre-drying before artificial drying	Artificial drying
A	1, 2, 3	Plastic bags during 1. night (d0-d1), then **jute bags** during nights	Sun-drying during day	Sun-drying during day, considering also as “turning-step”	Sun-drying during day	Directly in a small dryer without ventilation, close contact to the heat source
B	1	**Plastic bags**	No	Turning at d3 while placenta removement	1 day before artificial drying (last night in plastic bags)	Directly in dryer with ventilation, away from direct heat source (dependent repetitions separated by jute bags)
	2	**Plastic bags**	No	No		
C	1, 2, 3	**Plastic bags**	No	No	1 day before artificial drying (last night in switched-off dryer)	Directly in dryer with ventilation, away from direct heat source (dependent repetitions separated by wooden sticks)
D	1	Plastic bags during 1. night, then **jute bags**	Pre-drying at d1	No	Same day as artificial drying	Directly in the dryer with ventilation (dependent repetitions separated by jute bags)
	2	Jute bags during 1. night, then **jute bags**	No	No	Same day as artificial drying	In jute bags on top of other beans (with ventilation)
	3	Plastic buckets during 1. night, then **jute bags**	Pre-drying at d0	No	Same day as artificial drying	
E	1W, 2W	**Wooden box** (beans uncovered)	No	No	Same day as artificial drying	In jute bags on top of other beans (with ventilation)
	1P, 2P	**Plastic bags**	No	No

**Figure 4:**
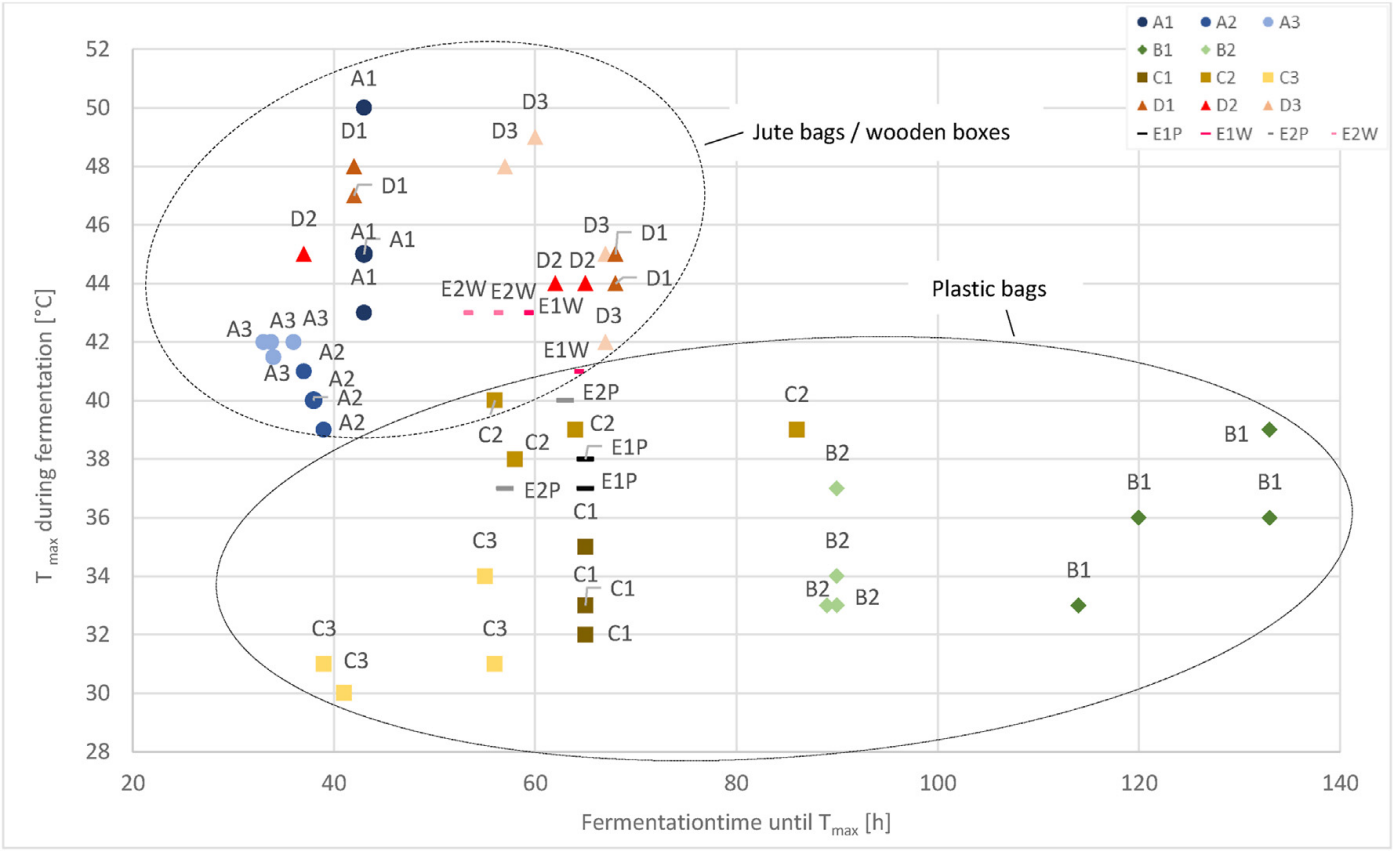
Reached maximum temperature (T_max_, [°C]) during fermentation and time until reaching T_max_ [h] at the five locations A-E (A1 as dark blue circle, A2 as mid blue circle, A3 as light blue circle; B1 as dark green rhombus, B2 as light green rhombus; C1 as dark gold square, C2 as mid gold square, C3 as yellow square; D1 as dark orange triangle, D2 as red triangle, D3 as light red triangle; E1P (plastic bags) as black and E2P (plastic bags) as grey linkes; E1W (wooden boxes) as dark pink and E2W (wooden boxes) as light pink line). During each run, 2–4 dependent repetitions were done and shown; Samples divided into fermentation in jute bags or wooden boxes (rough dashed line) and fermentation in plastic bags (fine dashed line).

Significantly higher maximum temperatures (Tmax) (p < 0.05) were reached in jute bags (location A, D) and wooden boxes (EW) than in plastic bags (locations B, C, and E (EP)) (Figure 4). At A, Tmax of 42–50 °C were reached after 33–43 h (A1 and A3) and 40 ± 1 °C after 37–39 h (A2). At D, slightly lower Tmax of 42–49 °C were reached after 37–68 h. Significant lower Tmax (p < 0.05) were measured at locations B and C with 30–40 °C after 88–133 h at B and 38–86 h at C. At location E, Tmax in wooden boxes with 43 ± 1 °C was significantly higher (p = 0.0034) than in plastic bags with 38 ± 2 °C. Tmax was reached after 60 ± 4 h (no significances between EW and EP, p = 0.2).

**Figure 5:**
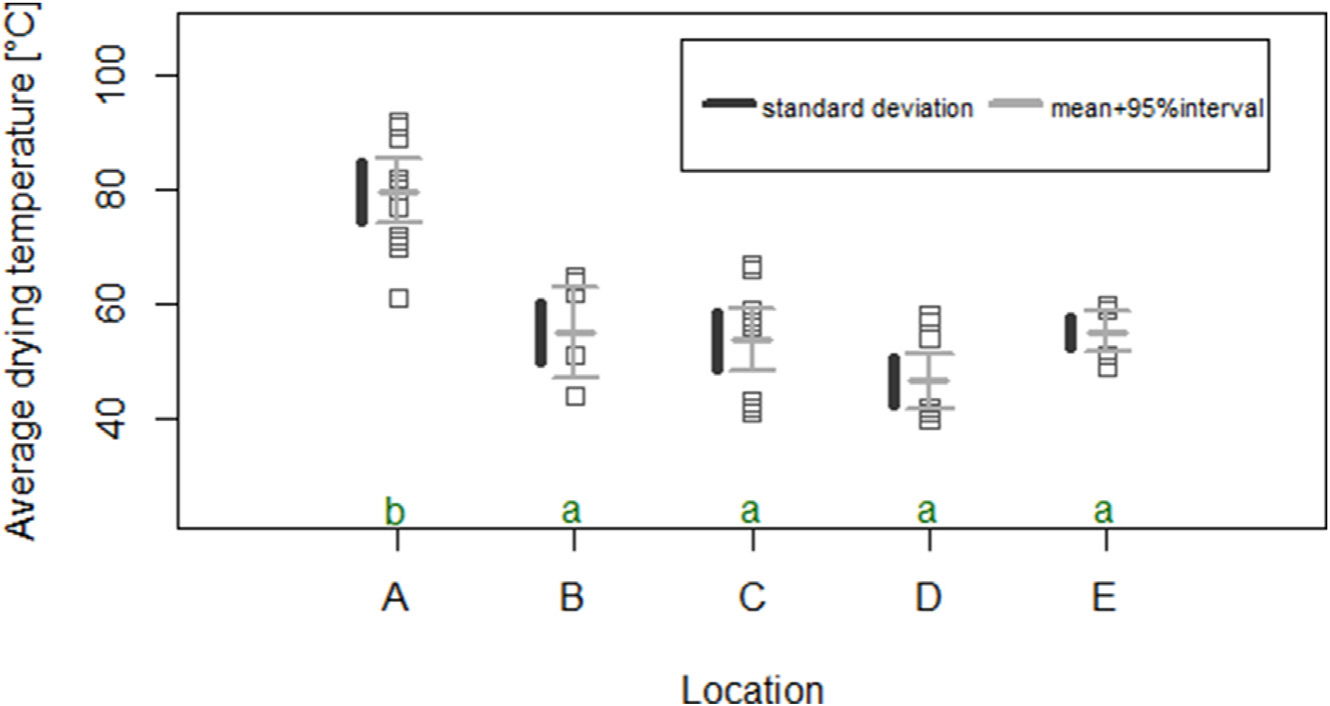
Average drying temperature [°C] with standard deviation (sd) and mean value at the five locations A (n = 12), B (n = 6), C (n = 12), D (n = 11), E (n = 8) (ANOVA with Tukey HSD); Average drying temperature that do not share the same letter differ significantly (p < 0.05).

During drying, varying set-ups and temperatures were applied. While the beans at location A were dried in small gas-powered dryers with close contact to the fire (no ventilation), beans at D (D2, D3) and E were dried in jute bags on top of other beans (with ventilation), and D1 was dried directly in the dryer with ventilation. At locations B and C, the beans were dried in a dryer with ventilation, away from direct heat source (dependent repetitions were separated by jute bags at B and by wooden sticks at C). Significant higher average drying temperatures were reached at location A (p < 0.05) with 80 ± 10 °C than at the other locations (Figure 5). The significant longest drying was needed at location D (p < 0.05). Nevertheless, temperatures of up to 110 °C (at location A), 102 °C (B), 114 °C (C), 125 °C (D), and 97 °C (E) were reached at certain points during the drying.

### 3.2 The succession of yeast, LAB, and AAB

General trends of microbial succession during post-harvesting were recognized but with several outliners. Initial yeast concentration was at all locations between 4.6 ± 0.6 (B1) and 7.1 ± 0.2 log cfu/g (D2) and increased within the first 24 h except in run D1, where a slight decrease was observed. Maximum counts of 7.4 ± 0.3 (D2) and 7.0–7.2 (E2) log cfu/g were reached already after 24 h in D2 and E2 (EP, EW), whereas in the majority of fermentation maxima were determined after 48 h: 6.7–7.7 log cfu/g (A1-A3), 7.0 ± 0.3 log cfu/g (B2), 7.3 ± 0.2 log cfu/g (C1), 6.50 ± 0.04 (D1), 7.6 ± 0.1 log cfu/g (D3), and 6.9–7.4 log cfu/g (E1) followed by a decrease until the end. The maximum yeast counts in the other runs were reached after 72 h in C3 with 7.6 ± 0.1 log cfu/g, after 96 h 7.0 ± 0.4 log cfu/g in C2 and after 120 h with 7.8 ± 0.1 log cfu/g in B1.

**Figure 6:**
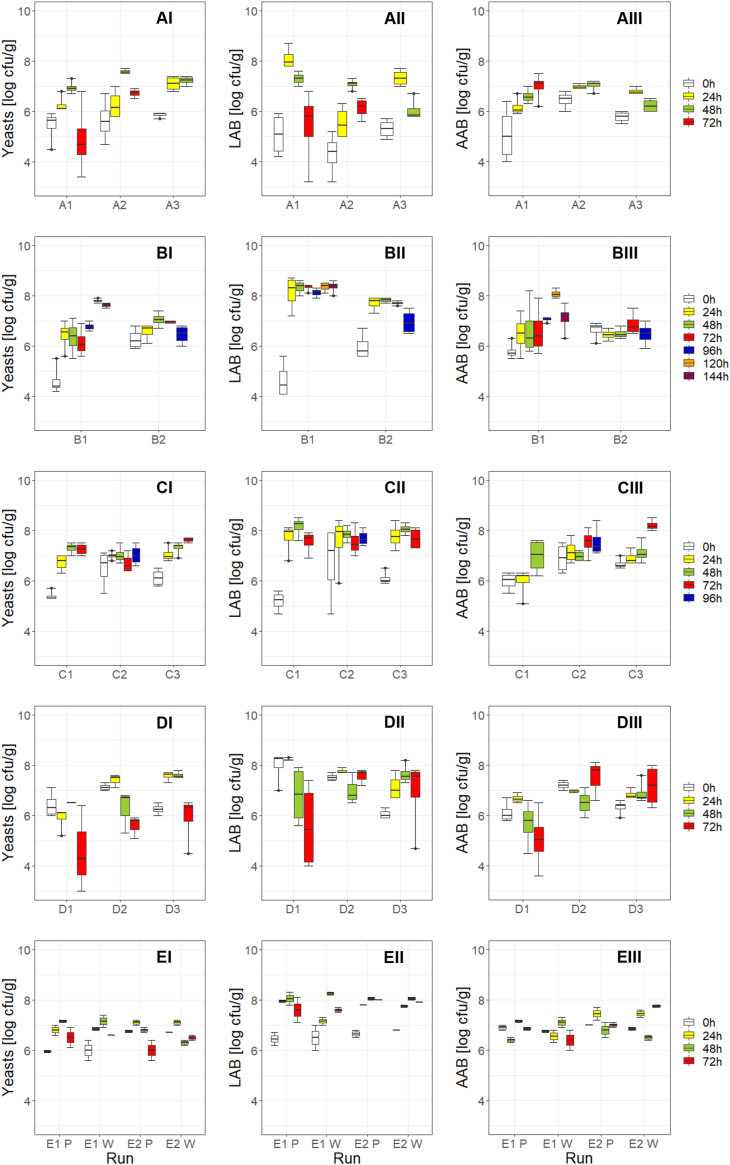
Plate counts [log cfu/g] of yeast (YGC, I), lactic acid bacteria LAB (MRS, II), and acetic acid bacteria AAB (YPM, III) at the five locations A (run A1, A2, A3; n = 4 each), B (run B1, B2; n = 4 each), C (run C1, C2, C3; n = 4 each), D (run D1, D3; n = 4 each and D2; n = 3), and E (run E1P, E1W, E2P, E2W; P for plastic bags, W for wooden boxes; n = 2 each) after 0 (white), 24 (yellow), 48 (green), 72 (red), 96 (blue), 120 (orange), and 144 h (claret) of fermentation; Location D (run D1): Yeast and AAB after 96 h = n. a., LAB after 96 h = 2.6 ± 0.9 log cfu/g (data not shown).

LAB at 0 h were between 4.3 ± 0.8 (A2) and 8.0 ± 0.7 (D1) log cfu/g (Figure 6). In four runs, LAB already increased to their maxima in the first 24 h with 8.1 ± 0.4 (A1), 7.3 ± 0.3 (A3), 8.2 ± 0.1 (D1), and 7.8 ± 0.1 (D2) log cfu/g. In all other fermentations maxima were reached after 48 h with comparable counts at locations B, C, E, and D (D3) in a range of 7.6 ± 0.4 (D3) to 8.2 (E1W) log cfu/g. Maximum LAB count in run A2 was lower with 7.1 ± 0.2 log cfu/g. After having reached their maxima, LAB decreased or stayed stable until the fermentation end.

Initial AAB concentrations were between 5.1 ± 1.1 (A1) and 7.2 ± 0.2 (D2) log cfu/g. In eight runs (A1, A2, C1, C2, C3, D2, D3, E2W), maximum concentrations were determined at the fermentation end with 7.0 ± 0.6 in A1, 7.0 ± 0.2 in A2, and 7.2 ± 0.9 in D2 after a slight increase from the beginning to the end, and 7.0 ± 0.7 log cfu/g in C1 and 8.2 ± 0.2 in C3 after a strong increase in the last 24 h. In C2 and E2W maximum counts were 7.5 ± 0.6 (C2) and 7.8 log cfu/g (E2W), respectively, after a fluctuating development and in D2 the maximum counts were 7.5 ± 0.8 log cfu/g after a decreasing trend from 0-48 h. In A3, D1, and E2P, AAB reached their maxima already after 24 h with 6.8 ± 0.1 in A3, 6.7 ± 0.2 in D1, and 7.4 log cfu/g in E2P. In B1, B2, E1P, and E1W, AAB reached their maxima at the day before the fermentation end, with 8.1 ± 0.2 cfu/g in B1, 6.9 ± 0.4 cfu/g in B2, 7.2 cfu/g in E1P, and 7.1 log cfu/g in E1W with a fluctuating development.

### 3.3 Species diversity during cocoa bean fermentations

MALDI-TOF MS analyses resulted in the identification of total 322 isolates (24.76% of 315 of the isolates from YGC agar, 32.21% of 267 re-cultivated isolates on MRS (11 isolates could not be re-cultivated in Switzerland), and 54.12% of 292 on YPM (1 isolate could not be re-cultivated)) (Table 3).

**Table 3:** Numbers of strains (yeasts, LAB, AAB, other) isolated during 2–3 independent runs and 2–4 dependent repetitions at the five locations A, B, C, D, E. Microorganisms were isolated during the fermentation days 0–6 and identified by MALDI-TOF MS (total 322 identified microorganisms from YGC, MRS, and YPM agar).

Species	Fermentation time [d]													
	0		1		2		3		4		5		6	
**Yeasts (total)**	**22**		**26**		**11**		**10**		**8**		**1**		**4**	
*Candida humilis*	-		1		-		-		-		-		-	
*Candida intermedia*		(1)		(1)	-		-		-		-		-	
*Candida krusei*	-		-		1		-		2		-		4	
*Candida parapsilosis*		(1)	-		-		-		-		-		-	
*Hanseniaspora opuntiae*	2	(14)	2	(14)		(4)		(1)	1		-		-	
*Hanseniaspora uvarum*	1		-		-		-		-		-		-	
*Kodamaea ohmeri*	-			(1)	-		-		-		-		-	
*Pichia kluyveri*		(1)	-		-		-		-		-		-	
*Pichia occidentalis*	-		1		-		1	(1)	1		-		-	
*Saccharomyces cerevisiae*		(2)		(6)		(6)		(6)		(4)	1		-	
*Trichosporon asahii*	-		-		-			(1)	-		-		-	
**Lactic acid bacteria (total)**	**22**		**20**		**18**		**22**		**3**		**0**		**1**	
*Filifactor villosus*		(2)		(1)	-		-		-		-		-	
*Lb. amylovorus*	-		-		-		1		-		-		-	
*Levilactobacillus brevis*	1	(2)	1	(1)	-		1	(4)			-		-	
*Limosilactobacillus fermentum*		(2)		(2)		(5)		(4)		(1)	-			(1)
*Liquorilactobacillus mali*	-		-			(1)	-		-		-		-	
*Lacticaseibacillus paracasei*	-		-		-		1		-		-		-	
*Lactiplantibacillus paraplantarum*	1		-		-		-		-		-		-	
*Lactiplantibacillus plantarum*	4	(4)	4	(6)	3	(9)	7	(4)	1	(1)	-		-	
*Paucilactobacillus suebicus*		(1)		(1)	-		-		-		-		-	
*Leuconostoc pseudomesenteroides*		(5)		(4)	-		-		-		-		-	
**Acetic acid bacteria (total)**	**7**		**23**		**48**		**32**		**9**		**5**		**1**	
*Acetobacter cerevisiae*	-		-		-			(1)	-		-		-	
*Acetobacter fabarum*	2		4	(1)	7	(1)	4	(1)	4		1		-	
*Acetobacter ghanensis*	1		9	(1)	16		7		-		-		-	
*Acetobacter indonesiensis*	-				1		1		-		1		-	
*Acetobacter lovaniensis*	-		1		-		-		-		-		-	
*Acetobacter orleanensis*	-		-		-			(1)	-		-		-	
*Acetobacter persici*	-		1		2	(2)		(2)	-		-		-	
*Acetobacter senegalensis*	-			(2)	3	(6)	1	(11)	1	(2)		(1)	-	
*Acetobacter syzygii*	-		-		2		-		-		-		-	
*Acetobacter tropicalis*	-			(1)		(2)	1	(2)		(2)	2		1	
*Gluconobacter oxydans*	1	(3)	2	(1)	4	(2)	-		-		-		-	
**Others (total)**	**14**		**9**		**2**		**1**		**1**		**0**		**2**	
*Aeromonas media*	-		-			(1)	-		-		-		-	
*Pseudomonas monteilii*	1		-		-		-		-		-		-	
*Enterobacter aerogenes*	-			(1)	-		-		-		-		-	
*Enterobacter cloacae*	2		-		-		-		-		-		1	
*Klebsiella oxytoca*	-		-		-		-		-		-		-	
*Klebsiella pneumoniae*		(1)	1	-	-		-		1		-		-	
*Pantoea agglomerans*		(1)	-		-		-		-		-		-	
*Raoultella terrigena*	-		-		-		-	(1)	-		-		-	
*Serratia marcescens*	1		1	-	-		-		-		-			(1)
*Staphylococcus capitis*	-		-		1		-		-		-		-	
*Tatumella citrea*		(1)	1		-		-		-		-		-	
*Tatumella ptyseos*	1	(2)	3	(1)	-		-		-		-		-	
*Tatumella punctata*	2	(1)		(1)	-		-		-		-		-	
*Tatumella terrea*		(1)	-		-		-		-		-		-	

Isolates are listed according to their identification regular numbers (MALDI score >2.000; secure genus identification, probable species identification) and numbers in brackets (MALDI score 1.700–1.999; probable genus identification).

*Hanseniaspora opuntiae* dominated the beginning of fermentation within the identified yeasts until day 1, followed by *Saccharomyces cerevisiae* dominating from day 2–4. *Lactiplantibacillus plantarum* was the dominant LAB species from day 0–4, *Leuconostoc pseudomesenteroides*, which was identified five and four times on day 0 and day 1, respectively, was the second most, and *Limosilactobacillus fermentum* was present during the whole fermentation (day 0–4 and 6). Of the 48 identified isolates at day 2, the dominant AAB species was *Acetobacter ghanensis* whereas at day 3 *Acetobacter senegalensis* dominated. AAB diversity increased from three species (*A. fabarum, A. ghanensis, Gluconobacter oxydans*) at day 0 to seven and eight species at day 1 and day 2/3, respectively (Table 3).

### 3.4 pH values of pulp and cotyledon

**Figure 7:**
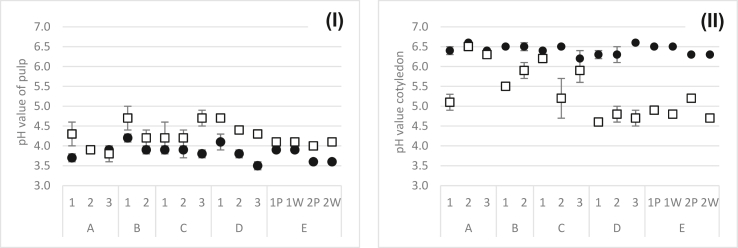
PH of pulp (I) and cotyledon (II) at the start (black dots) and end (white squares) of fermentation at the five locations A (run A1, A2, A3; n = 4 each), B (run B1, B2; n = 4 each), C (run C1, C2, C3; n = 4 each), D (run D1, D3; n = 4 each and D2; n = 3), E (run E1P, E1W, E2P, E2W; P for plastic bags, W for wooden boxes; n = 2 each).

Pulp pH at day 0 was similar at the five locations and between 3.5 ± 0.1 (average measured in D3) and 4.0 ± 0.1 (average in C1) with two outliers, 4.1 ± 0.2 in D1 and 4.2 ± 0.1 in B1. Pulp pH at locations B, C, and E increased in the first 24 h, followed by a de- and increase in the last 48 h (B1, C2) or 24 h (B2, C1, C2 and marginal increase in E) to the following values: 4.7 ± 0.3 (B1), 4.2 ± 0.2 (B2), 4.2 ± 0.4 (C1), 4.2 ± 0.2 (C2), 5.0 ± 0.2 (C3), and 4.0–4.1 (E). Constantly increasing pulp pH to 4.3 ± 0.3 (A1) and 4.38 ± 0.03 (D2) was measured in A1 and D2. In A2 it was almost stable until a marginal increase to 3.9 ± 0.1 in the last 24 h. In D3, pulp pH increased until the last 24 h and subsequently decreased to 4.3 ± 0.1. A3 and D1 showed an initial decrease of pulp pH for 24 h and increased then to 3.8 ± 0.2 (A3) and to 4.7 ± 0.1 (D1). Notable differences of pulp pH between day 0 and fermentation end were observed in A1, B1, C3, D1, D2, D3, E2P, E2W (Figure 7I).

The initial pH of cotyledon was between 6.2 ± 0.2 (average measured in C3) and 6.60 ± 0.03 (average in A2) at all locations and decreased steadily in A1, D2 (but both almost stable for the first 24 h), A2, A3, B1, C1 and E2 (both almost stable with an increasing tendency in the last (C1) or first (E2) 24 h), D1, D3, and E1. A slight increase during the first 24 h, followed by a subsequent de- and a slight increase within the last 24 h was observed in C3. In B2 and C2 cotyledon, pH dropped before a slight increase within the last 24 h. Marginal differences from day 0 to fermentation end were observed in A2 (end pH cotyledon was 5.1 ± 0.2), A3 (6.45 ± 0.03), B2 (5.9 ± 0.3), and C1 (6.2 ± 0.1) (Figure 7II).

### 3.5 Cut-test of dried cocoa beans

**Figure 8:**
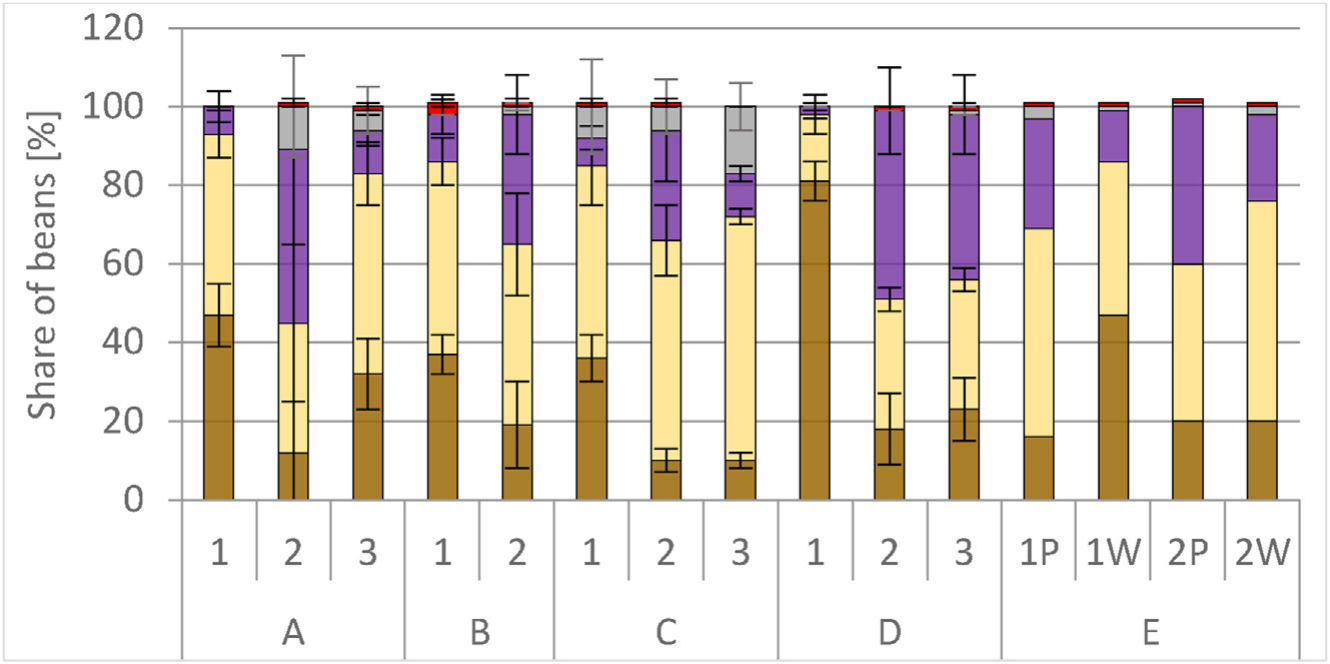
Cut-test performed with 100 dried beans of locations A (run A1, A2, A3; n = 4 each), B (run B1, B2; n = 4 each), C (run C1, C2, C3; n = 4 each), D (run D1, D3; n = 4 each and D3; n = 3) and E (run E1P, E1W, E2P, E2W; P for plastic bags, W for wooden boxes; n = 2 each). The beans were classified as well-fermented (brown), slightly fermented (yellow), violet (violet), slaty (grey), or mouldy (red).

At each location, cut-tests reached in minimum in one run >80% total fermented beans (Figure 8). Samples of runs A1 and D1 reached the highest averages with 93 ± 5% (A1) and 98 ± 3% (D1). High shares of violet beans were present in A2 with 44 ± 24% (significantly more than in A1 and A3, p < 0.05), D2 (48 ± 11%), and D3 (42 ± 10%), the latter being significantly higher than determined in D1 (p < 0.05). Significantly higher amounts of slaty beans (p < 0.05) were observed in all samples at location C (highest value 17 ± 6% in the sample of C3), compared to B, D, E. At A, samples of A2 showed on top a high standard deviation of 11 ± 13% for slaty beans. Significant highest amounts of moldy beans were found (3 ± 1%, p < 0.05) in B1. Beans attacked by insects or worms were not found in any of the samples.

### 3.6 Sensory description of the cocoa bean liquor

The twelve mixed samples per run were discriminated with eleven out of the 13 defined sensory attributes (average ratings and p-values in Table 4).

**Table 4:** Sensory description by QDA: Attributes (Mixed ANOVA with Panelist and Panelist:Sample as random and Sample as fixed factor) and average scores (significant attributes in bold); samples from the five locations A (run A1, A2, A3), B (run B1, B2), C (run C1, C2, C3), D (run D1, D2, D3), E (run E1, E2).

Sensory attribute	p-value	A1	A2	A3	B1	B2	C1	C2	C3	D1	D2	D3	E1	E2
Acidity	**0.000**	4.6	1.4	1.4	3.4	2.0	1.3	3.5	2.0	7.7	7.6	7.4	7.0	7.0
Bitterness	**0.000**	2.5	5.9	6.0	4.1	5.2	5.8	3.9	6.9	2.1	2.8	2.0	2.5	2.3
Fruity:banana	**0.002**	1.1	0.7	0.8	0.3	0.3	0.7	1.2	0.6	1.8	2.6	3.1	0.7	2.0
Fruity:citrus	**0.000**	1.1	0.2	0.4	0.3	0.6	0.5	1.7	0.4	4.0	3.5	4.2	4.7	3.3
Floral	0.162	1.1	1.3	1.8	0.4	2.8	2.1	2.3	2.8	1.0	2.0	1.5	2.8	2.2
Nutty	**0.000**	2.4	4.3	2.0	2.1	3.4	3.4	3.2	3.4	1.1	1.2	0.9	2.4	1.4
Woody	**0.000**	2.8	6.5	3.6	2.5	4.3	5.6	3.3	6.0	0.9	1.9	1.5	1.7	1.8
Spicy	0.857	3.2	2.3	1.9	2.7	1.8	2.4	1.9	2.9	2.5	1.9	1.7	2.1	2.6
Malty	**0.006**	1.4	0.5	0.7	0.2	0.4	0.3	0.2	0.3	1.2	0.4	0.5	0.4	0.5
Roasted-Burnt	**0.000**	5.2	2.8	6.1	1.2	1.7	3.5	1.4	2.9	3.1	0.7	2.6	1.7	2.9
Earthy/Mouldy	**0.000**	0.7	0.7	0.5	7.6	0.4	0.3	0.6	0.5	0.5	0.5	0.4	0.4	0.5
Chemical	**0.000**	0.6	0.8	0.7	2.2	0.3	0.2	0.5	0.2	1.6	3.3	1.7	0.3	0.8
Astringency	**0.000**	2.2	4.4	3.9	2.4	3.1	5.1	2.7	4.7	2.0	2.3	2.6	2.4	1.8

**Figure 9:**
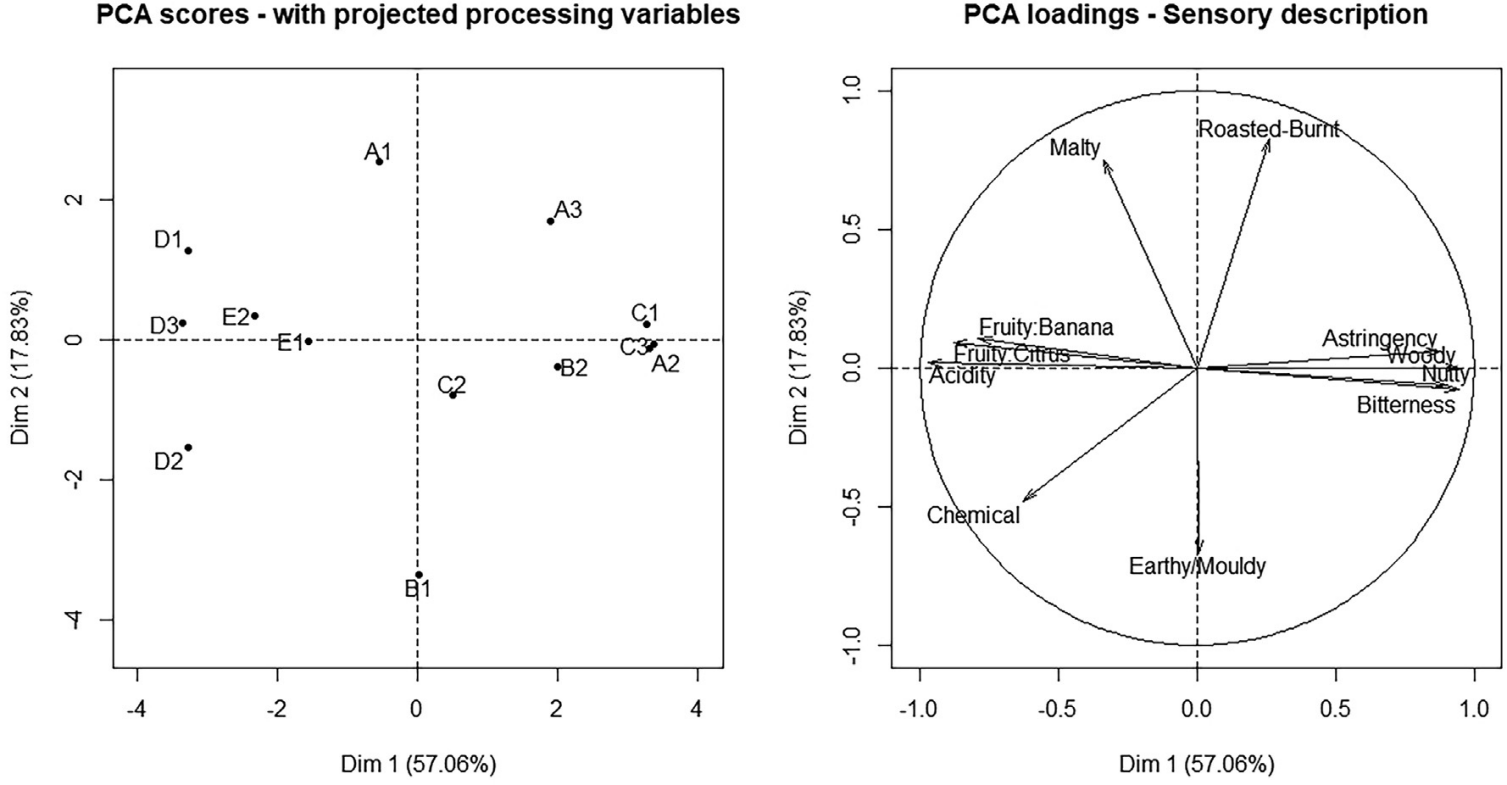
Principal component analysis (PCA) of significant standardized sensory attributes from cocoa liquor samples from locations A (run A1, A2, A3; n = 1 each), B (run B1, B2; n = 1 each), C (run C1, C2, C3; n = 1 each), D (run D1, D2, D3; n = 1 each), E (run E1, E2; n = 1 each).

A PCA (Figure 9) with the significant flavor attributes revealed a first component where the attributes acidity, fruity:banana, fruity:citrus, and chemical correlated negatively to bitterness, astringency, nutty, and woody and a second component representing off-flavors earthy/moldy, chemical as well as roasting flavors malty and roasted-burnt.

Beans at locations D and E resulted in more fruity-acidic cocoa liquor and at A, B, and C in more bitterness and astringency, dominating cocoa liquor at different degrees. Samples A2, A3, B2, C2, and C3 were more pronounced in these attributes than A1, B1, and C1, which were placed in the center of the first component. B1 was rated high regarding the off-flavors earthy/moldy and chemical. A chemical off-flavor was also increased for D2. Further, A1 and D1 were rated high regarding the attribute malty indicating roasting flavors, and for A1 and A3, the attribute roasted-burnt was high, indicating a burnt off-flavor.

## 4 Discussion

### 4.1 Development of process parameters during exemplary post-harvest processing in Ecuador

In this study, post-harvest techniques applied by Ecuadorian intermediaries were investigated. Bags (jute or plastic) were recognized as the most common fermentation device, even though they were not recommended by Teneda Llerena (2016) due to the risk of poor aeration and high humidity resulting in a high percentage of violet and slaty beans. However, the literature currently lacks detailed studies on fermentation in plastic bags.

Investigated fermentations lasted 74 ± 21 h, with the shortest duration (46 h) at location A (A3) and the longest (133 h) at B (B1) and in average below the recommended 120–144 h during fermentation in, e.g., wooden boxes (Papalexandratou et al., 2011b). We recognized 67–74 h for jute bags, as at location D, as sufficient for satisfactory bean quality (fruity-acidic and neither astringent, woody, nutty, nor bitter cocoa liquor). Post-harvest processing at A with short fermentation in jute bags for 64 h even resulted in 93 ± 5% (A1) total fermented beans with an intermediate acidity-bitterness/astringency profile accompanied however by a burnt off-flavor, which might have resulted from significant higher average drying temperature and the dryer itself (direct heat contact without ventilation). This shows that even a shorter fermentation carried out in jute bags with subsequent drying at high average drying temperatures, might be sufficient to reach well-fermented cocoa beans. This leads to the assumption that high drying temperatures might falsify the cut-test.

Similar to our study, previous studies showed influences of the device on the temperature (e.g. Quevedo Guerrero et al., 2018; Castañeda Chávez et al., 2020). Typical temperature developments up to 42–50 °C (A1, A3) and 42–49 °C (D1-D3) were observed in jute bags. In run A2 lower temperatures (39–42 °C) were reached, probably resulting in an incomplete fermentation and in a high variation in cut-test result within dependent repetitions, which could be seen by the high amount and high standard deviation of violet (44 ± 24%) and slaty (11 ± 13%) beans, and which could be explained by the nutty taste of the liquor that was stronger than of beans from A1 and A3. The combination of woody flavor (or “green notes”) and weak or little fermentation was similarly described by Luna et al. (2002). In this study, the nutty flavor was defined with unroasted nuts with or without skins, as well as raw cocoa beans (without pungent acidity) unlikely, as described in Afoakwa et al. (2008), where pyrazines, generated during roasting, cause the nutty flavor. In wooden boxes (EW), temperatures up to 43 ± 1 °C were measured. Interestingly, the temperature in plastic bags at location E (EP) were lower (38 ± 2 °C). In other plastic bags (locations B, C) only 30–40 °C (Tmax) were reached, which is clearly below known maximum values of 45–50 °C and during a rather long fermentation time of 62–133 h (Afoakwa, 2010; De Vuyst and Weckx, 2016; Fowler, 2009; Schwan et al., 2014). Better heat conservation in jute than in plastic bags can therefore be assumed.

Decreasing yeast counts while increasing the pulp-bean-mass temperature was observed as comparable to De Vuyst and Weckx (2016). In contrast, the temperature courses determined, e.g., with low temperatures in plastic bags at B and C, did not match the relatively high amounts of AAB, which should have contributed to an increased temperature (Afoakwa, 2010; De Vuyst and Weckx, 2016).

*H. opuntiae* was the predominant yeast species at the beginning of fermentation, followed by *S. cerevisiae*, which was also observed by Romanens et al. (2018). *Lactiplantibacillus plantarum* was the dominant LAB species identified from day 0–3, which was determined comparably by Papalexandratou et al. (2011b) in Ecuador. *A. ghanensis* as dominant AAB species from day 1–2 and *A. senegalensis* as dominant AAB species at day 3 matched with their occurrence described in previous studies (De Vuyst and Weckx 2016; Miescher Schwenninger et al., 2016; Romanens et al., 2018). As in the study of Papalexandratou et al. (2011b), *A. pasteurianus* was not identified in any of the fermentation*,* even though it counts to the dominating AAB species in cocoa bean fermentation (De Vuyst and Weckx 2016).

In this study, a wider initial (3.5 ± 0.1 to 4.2 ± 0.1) and end pulp pH (3.8 ± 0.2 to 4.7 ± 0.1) was observed than described by Papalexandratou et al. (2011b), which might be due to a broad number of different farms providing cocoa beans to the intermediaries going along with varying maturity of beans or pod storage prior to opening that influences initial pulp pH (Afoakwa et al., 2013), whereas natural differences in initial material and occurring microorganisms combined with the varying post-harvest treatments impact the end pulp pH. No significant differences of the delta (initial to end pH) between the devices were calculated (p > 0.05), but from location A (significantly lower) to location D (p < 0.05).

The pH development of the cotyledon is strongly related to metabolites (e.g., lactic, acetic acid) of microbial activity diffusing into the beans (Schwan and Wheals, 2004). In this study, initial pH values of cotyledon were 6.2 ± 0.2 to 6.60 ± 0.03 and thus slightly lower than described by Papalexandratou et al. (2011b). It decreased to 4.6 ± 0.1 to 6.45 ± 0.03 at the fermentation end, which was clearly above the 4.21–4.24 monitored by Papalexandratou et al. (2011b). Probably, differences observed are related to variations in metabolic activity during fermentation. Here, the device seemed to influence the cotyledon pH. From start to fermentation end, the pH development in cotyledon in plastic bags was significantly lower than in wooden boxes (p < 0.05).

A negative correlation was found between end cotyledon pH and Tmax during fermentation: The lower Tmax, the higher the end cotyledon pH. This phenomenon was clearly visible at locations B and C, where plastic bags were used, going along with the assumption of Thompson et al. (2001) that no or only weak temperature increase is concomitant with lower organic acid production and therefore does not result in a decreased pH. Additionally, significantly more organic acids can be produced depending on the presence of microorganisms, which reflects, e.g., temperature and pH development and the available substrates (Moreira et al., 2013). However, acids were not analyzed in this study, but the temperature seems to indicate the quantity of produced microbial metabolites diffusing into the cotyledon and lowering the pH.

Regarding the drying process, the most significant difference was the contact of the beans to the heat source, the temperature reached at certain points >100 °C, which is beyond the recommended 60 °C (Thompson et al., 2001).

### 4.2 Influence of process parameters on quality of dried cocoa beans

A significant negative correlation was observed between Tmax during fermentation and the amount of slaty (r = -0.42, p < 0.05) and slightly fermented (r = -0.47, p < 0.05) beans, and a significant positive correlation with well-fermented beans (r = 0.41, p < 0.05). This matches the description of internal enzymatic and external microbial processes of cocoa beans with rising temperature up to 50 °C and microbial built ethanol and acetic acid that are responsible for the seed death (Ardhana and Fleet, 2003; Camu et al., 2008; Lima et al., 2011), followed by the release of enzymes which start an array for the flavor formation important endogenous biochemical reactions (Ardhana and Fleet, 2003; Guehi et al., 2010). As a result of these complex reactions during fermentation, the violet color of the bean changes to brown (Fowler, 2009). Slaty, slightly fermented, and violet beans indicate an absence or insufficient fermentation (Fowler, 2009; Guehi et al., 2010) and Tmax during fermentation could thus be a direct indicator to the color change. Similarly, the insufficient fermentation with slaty and only slightly fermented beans was reflected on the liquor flavor and a high ratio correlated in bitter and astringent cocoa samples, which was also concluded by Guehi et al. (2010). On the other hand, fruity-acidic samples correlated with a higher ratio of well-fermented beans, whereas slightly fermented beans were negatively correlated to acidity, fruity:banana, fruity:citrus, malty and chemical. In earlier studies, fermentation time or device did not influence the content of slaty beans and rather a relation to the degree of ripeness of the fruits than to the fermentation was hypothesized (Castañeda Chávez et al., 2020; Rivera Fernández et al., 2012).

Cocoa liquors produced from locations D and E were more fruity-acidic, probably due to the device (jute bags, wooden boxes) and the corresponding temperature development combined with the time and therefore a more completed fermentation process, while cocoa liquor from A, B, and C were more bitter and astringent which can be explained by the not properly running fermentation process due to plastic bags (locations B, C) or the short fermentation time (A).

One specific fermentation of location B (B1) resulted in beans with moldy off-flavor corresponding to an increased ratio of moldy beans in the cut-test and correlated to a long fermentation time (supplementary material, Fig. A1). An outgrowth of molds was probably favored by the long fermentation time in plastic bags concomitant with a rather high end cotyledon pH and rather low fermentation temperatures (36 ± 2 °C in B1) as stated by Schwan and Wheals (2004), who described that molds are mostly found in cooler and superficial areas of the fermenting mass. Further, a high average drying temperature had a significant impact on the amount of violet beans (p < 0.05), which was observed at locations A (A1, A3 but not in A2) and D (D1). This might indirectly influence the interpretation of the development of fermentation, but the assumption of falsifying the cut-test due to the drying temperature should be considered. The high temperatures also influenced the flavor: Beans at location A had a roasted-burnt off-flavor (significant correlation, p < 0.05). Generally, it is agreed that rapid drying affects the quality of the beans negatively, as reviewed by Amoa-Awua (2015) and should not exceed 60 °C (Thompson et al., 2001).

A pH of cotyledon at the last day of fermentation <5, e.g., the average per fermentations at D and E, led to significantly higher acid, fruity:banana, fruity:citrus and lower bitter, nutty, woody, and astringent beans. This effect can be explained with the increasing and diffusing microbial built acids, including acetic acid, into the cotyledon (Camu et al., 2008; Thompson et al., 2001) and, concomitantly, a reduction of the bitterness and astringency through the oxidation of polyphenols during the fermentation (Kongor et al., 2016).

Sensory indicators of the fermentation development were responsible for the main differentiation of the samples. The fermentation development was defined to come from bitterness and astringency to acidity, meaning that the attribute acidity was indicating a further developed fermentation concomitant with a better fermentation due to probable diffusion of the microbial built acids into the cotyledon. Bitterness and astringency that are correlated to polyphenol contents represented on the contrast a low development and therefore a weak fermentation. Polyphenols are responsible for astringent, bitter, and green notes (Luna et al., 2002), and their concentration decreases during fermentation due to polymerization, oxidation and interactions with proteins (Sabahannur et al., 2018).

## 5 Conclusion

To the best of our knowledge, this study covers the first monitoring of a broad variety of cocoa post-harvest practices in Ecuador at a small-farm level. It included the documentation of the process accompanied by the measurement of process variables, microbiological analysis, and the evaluation of dried beans. An inconsistency in processing was observed at the five different locations in pre-drying and turning steps during fermentation, which might also have influenced the process and the end-product. In this study, no statement about pre-drying and turning is possible. Also other aspects as for example pod storage or mechanical depulping were not investigated. Therefore, further studies focusing on these process steps are necessary. Further, the use of different devices (jute/plastic bags, wooden boxes) significantly impacted the process and the end-product. Higher temperatures were reached in wooden boxes and jute bags than in plastic bags, which might be explained by better heat conservation in jute than in plastic bags. As in wooden boxes, a typical temperature increase was observed during fermentation in jute bags, whereas fermentation in plastic bags generally led to rather low temperatures. Higher Tmax were associated to a higher degree of well-fermented beans as well as more acidic and fruity and less bitter and astringent flavors in the sensory description. Drying at high temperatures led to burnt off-flavors. Conclusively, fermentations in plastic bags seemed not suitable, while jute bags could be an alternative to wooden boxes. Both fermentation and drying were crucial to reach well-fermented beans without off-flavors. The findings of this study are a first step towards a post-harvest process optimization at a small-scale level.

## Declarations

### Author contribution statement

Stefanie Streule: Conceived and designed the experiments; Performed the experiments; Analyzed and interpreted the data; Wrote the paper.

Susette Freimüller Leischtfeld: Conceived and designed the experiments; Analyzed and interpreted the data; Wrote the paper.

Martina Galler: Performed the experiments; Analyzed and interpreted the data; Contributed reagents, materials, analysis tools or data; Wrote the paper.

Susanne Miescher Schwenninger: Conceived and designed the experiments; Wrote the paper.

### Funding statement

This work was supported by the Lindt Chocolate Competence Foundation.

### Data availability statement

Data included in article/supp. material/referenced in article.

### Declaration of interests statement

The authors declare no conflict of interest.

### Additional information

No additional information is available for this paper.

## Appendix A Supplementary data

Streule et al_Supplementary material_20220523_Rev5 Streule et al_Supplementary material_20220523_Rev5
