# Supplementary material for: Monitoring of cocoa post-harvest process practices on a small-farm level at five locations in Ecuador
Source: Heliyon. 2022 Jun 9;8(6):e09628. doi: 10.1016/j.heliyon.2022.e09628 (PMC9213719; doi:10.1016/j.heliyon.2022.e09628)
Supplement: Streule et al_Supplementary material_20220523_Rev5 [file mmc1.pdf]

Supplementary material

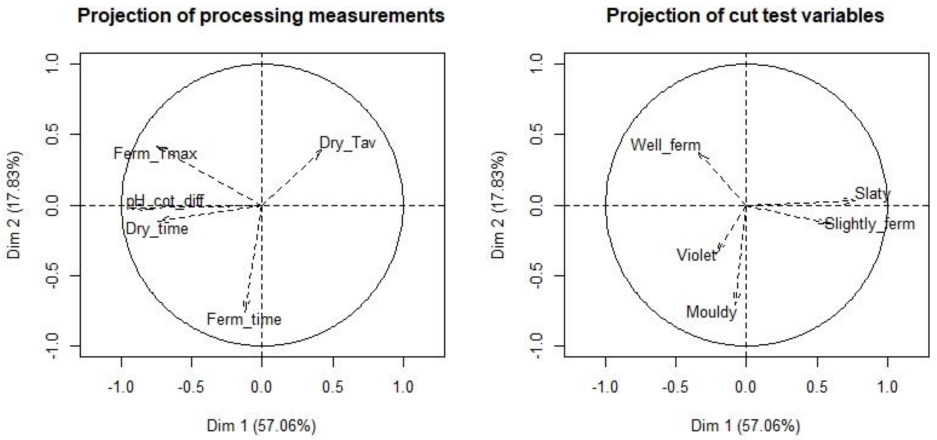

Fig. A.1 Projection of processing (Ferm_Tmax: Maximal fermentation temperature; Dry_Tav: Average drying temperature; Ferm_time: Fermentation time; Dry_time: Drying time; pH_cot_diff: Difference between initial and end pH in cotyledon) and cut test (Well_ferm: Well fermented beans; Slaty beans; Slightly_ferm: Slightly fermented beans; Mouldy and violet beans) variables on PCA of sensory description from Fig.9 (All variables were centered and standardized. The plot shows how variables were correlated in the presented explorative study. Note that factors related to location could have confound displayed correlations).
